# Factors influencing health-related quality of life in children with leukemia in Saudi Arabia: a cross- sectional study

**DOI:** 10.1186/s12887-026-07466-2

**Published:** 2026-08-08

**Authors:** Mohammed T. A. Omar, Hatem Mosa Maeshi, Amani A. Alkofide, Zizi M. Ibrahim, Mohamed Abdelaziz Emam, Maha F. Algabbani, Tahani H. Maashi, Olfat Ibrahim Ali, Mohammed A. Zalah

**Affiliations:** 1 Faculty of Physical Therapy, Cairo University, Giza, 12612 Egypt; 2 Department of Rehabilitation Health Sciencesm, College of Applied Medical Sciences, King Saud University, Riyadh, 11433 Saudi Arabia; 3 Ministry of Health, Riyadh, 11433 Saudi Arabia; 4 Consultant Pediatric Hematology Oncology, King Faisal Specialist Hospital & Research Center, Riyadh, Saudi Arabia; 5 Department of Rehabilitation Sciences, College of Health and Rehabilitation Sciences, Princess Nourah bint Abdulrahman University, P.O. Box 84428, Riyadh, 11671 Saudi Arabia; 6 János Szentágothai Neurosciences Division, Semmelweis University, Budapest, 1085 Hungary; 7 Basic Sciences Department, Faculty of Physical Therapy, Kafrelsheikh University, Kafr ash Shaykh, 33511 Egypt; 8 Consultant Pediatric Infectious Diseases, King Fahd Central Hospital, Jazan, 82511 Saudi Arabia; 9 Physical Therapy Program, Batterjee Medical College, Jeddah, 21442 Saudi Arabia; 10 Basic Science Department for Physical Therapy, Faculty of Physical Therapy, Cairo University, Giza, 12613 Egypt; 11 Medical Rehabilitation Center, King Fahd Central Hospital, Jazan, 82511 Saudi Arabia

**Keywords:** Health-related quality of life, leukemia, PedsQL™ 3.0 cancer module, Psycho metric assessment

## Abstract

**Background/objective:**

Leukemia and its treatment can substantially impair children’s health-related quality of life (HRQoL). This descriptive cross-sectional study aimed to evaluate HRQoL among Saudi children with leukemia and to identify the demographic, clinical, and functional factors associated with HRQoL.

**Methods:**

Eighty-six children aged 5–14 years with acute lymphoblastic or acute myeloid leukemia (AML) who were receiving active treatment at King Faisal Specialist Hospital and Research Center Riyadh, Saudi Arabia. All participants had been diagnosed at least three months before enrolment and were receiving active treatment. HRQoL was assessed using the Arabic version of the Pediatric Quality of Life Inventory™ (PedsQL™) 3.0 Cancer Module, while physical function was evaluated using the Quick Disabilities of the Arm, Shoulder and Hand (Quick-DASH) questionnaire and the Lower Extremity Functional Scale (LEFS). Correlations and theory-driven multiple linear regression analyses were performed to identify factors associated with HRQoL.

**Results:**

The mean overall HRQoL score was 62.7 ± 16.5, indicating a moderate level of HRQoL. The lowest scores were observed for procedural anxiety, whereas communication had the highest domain score. Girls demonstrated significantly higher HRQoL than boys.Multiple linear regression identified female gender (β = 0.270, 95% CI: 4.457–13.241, *p* < 0.001), older age (β = 0.229, 95% CI: 1.951–7.986, *p* = 0.002), higher educational level (β = 0.239, 95% CI: 1.933–7.437, *p* = 0.001), and better lower-extremity function measured by the LEFS (β = 0.307, 95% CI: 0.142–0.434, *p* < 0.001) as significant positive predictors of higher HRQoL, whereas more intensive cancer therapy was independently associated with lower HRQoL (β = −0.211, 95% CI: −8.808 to − 1.922, *p* = 0.003). The model explained 70.1% of the variance in HRQoL (R² = 0.701; adjusted R² = 0.665; F(9,76) = 19.753, *p* < 0.001).

**Conclusions:**

HRQoL in children with leukemia was independently associated with physical function, cancer treatment, and several sociodemographic characteristics. These findings highlight the importance of incorporating functional assessment and multidisciplinary supportive care into the management of children with leukemia to optimize HRQoL.

## Introduction

Leukemia is a malignant disorder of the blood-forming tissues and remains one of the most serious diseases threatening children’s lives and well-being [1]. In Saudi Arabia, leukemia is the most common childhood cancer, accounting for nearly 38.8% of all cancers among children under 14 years of age with a higher prevalence in boys than girls (59.6% vs. 40.9%) [2]The most frequently diagnosed types are acute lymphoblastic leukemia (ALL) and acute myeloid leukemia (AML), while chronic forms are extremely rare. Despite its high malignancy, remarkable advances in diagnosis and treatment have significantly improved survival rates over the past five decades. In Saudi Arabia, the five-year overall survival rate has reached 74.6%, which is lower than that reported in the United States, and remains favorable compared with survival outcomes reported in several other countries (54.9%–71.9%) [3–7]. However, increased survival comes at a cost. Children undergoing leukemia treatment often face a wide range of short- and long-term complications, including anemia, recurrent infections, pain, fatigue, muscle weakness, reduced cardiovascular fitness, diminished bone mineral density, and impaired balance and coordination [8]. Beyond these physical complications, many children also experience cognitive, emotional, and social problems, such as anxiety, depression, fear of recurrence, and uncertainty about the future [9–11].

Together, these factors can profoundly affect a child’s physical abilities and psychological adjustment, leading to reduced health-related quality of life (HRQoL). Evidence suggests that HRQoL may decline as early as the time of diagnosis and remain impaired for years after treatment completion [9, 10, 12, 13]. HRQoL is a subjective, multidimensional construct that reflects an individual’s perception of the effects of disease and treatment on physical, emotional, and social functioning [14]. Some models also include a spiritual dimension [15]. In recent years, HRQoL has become an essential outcome in pediatric oncology. Assessing HRQoL from the child’s perspective provides valuable insight into daily challenges and unmet needs. It also helps parents, healthcare professionals, and policymakers tailor interventions that address not only survival but also overall well-being [16]. Furthermore, HRQoL assessment can guide treatment modifications, support the development of rehabilitation programs, and inform families and healthcare professionals about the management of the long-term effects of leukemia and its treatment [8].

Alongside advances in medical care, there is growing recognition worldwide that the cancer journey involves not only curing the disease but also supporting children’s physical, social, and emotional well-being. Children with leukemia often experience reduced participation in school and play, impaired physical performance, and social isolation because of prolonged hospitalization and treatment-related fatigue [3, 17]. These experiences can also affect their families by increasing parental stress, disrupting family dynamics, and reducing caregivers’ quality of life [18]. It is therefore important to understand how treatment-related factors, family characteristics, and functional abilities interact to influence children’s quality of life. Studies suggest that improving functional outcomes through exercise and rehabilitation may also enhance emotional well-being and social interaction [19, 20]. These findings support the integration of HRQoL assessment into multidisciplinary cancer care so that improved survival is accompanied by meaningful recovery and a good quality of life. Despite increasing recognition of HRQoL as a critical component of cancer care, evidence from Arab countries remains limited. The few regional studies, including studies from Egypt [9, 10], Lebanon [11], and the Gaza Strip [12], have reported notable differences in HRQoL among children living with or surviving cancer. However, research specifically examining HRQoL among children with leukemia in Saudi Arabia remains scarce. Little is known about how demographic characteristics, treatment-related variables, and physical function jointly affect HRQoL in this population. Children undergoing leukemia treatment frequently experience musculoskeletal impairments, including muscle weakness, fatigue, reduced mobility, and limitations in upper- and lower-extremity function, which may interfere with daily activities and contribute to poorer HRQoL [13, 19, 20]. Few studies have assessed HRQoL alongside measures of upper- and lower-extremity function or examined the associations of demographic, treatment-related, and functional factors with HRQoL during active treatment. Assessing physical function using the Quick Disabilities of the Arm, Shoulder and Hand (QuickDASH) questionnaire and the Lower Extremity Functional Scale (LEFS) may provide a more comprehensive understanding of children’s functional status and its relationship with HRQoL. Therefore, this study aimed to assess HRQoL among children with leukemia in Saudi Arabia and to examine its associations with demographic, treatment-related, and physical-function variables using the PedsQL™ 3.0 Cancer Module, the QuickDASH questionnaire, and the LEFS. The findings may inform supportive-care and rehabilitation strategies designed to improve quality of life and daily functioning among children receiving treatment for leukemia.

## Materials and methods

### Study design and participants

This descriptive, cross-sectional, exploratory study used a convenience sampling method to recruit eligible children who attended the outpatient Oncology/Hematology Clinic at King Faisal Specialist Hospital and Research Center, in Riyadh, Saudi Arabia, during the study period. Children were eligibl if they were 5–14 years of age, had a confirmed diagnosis of with acute lymphoblastic leukemia (ALL) or acute myeloid leukemia (AML) for at least three months, were receiving active treatment, and could understand Arabic. Active treatment was defined as ongoing chemotherapy at the time of enrollment, irrespective of the treatment phase., irrespective of the treatment phase. Eligible children were invited to complete the study assessments. Children with musculoskeletal disorders, congenital deformities, terminal-stage disease, major developmental disorders, or other major comorbidities were excluded.

Ethical approval was obtained from the Institutional Review Board of King Faisal Specialist Hospital and Research Center in Riyadh (No. C380/350/42). The study objectives and data-collection procedures were explained verbally and in writing to the participants and their parents before written informed consent was obtained.

Ethical approval was obtained from the Institutional Review Board of King Faisal Specialist Hospital and Research Center in Riyadh (No. C380/350/42). The study was conducted in accordance with the ethical principles of the Declaration of Helsinki and complied with all applicable institutional and national ethical guidelinesThe study objectives and data-collection procedures were explained verbally and in writing to the participants and their parents before written informed consent was obtained.

### Sample size

The sample size was calculated using G*Power version 3.1.9.2 (Statistical Consulting Group, University of California, Los Angeles, CA, USA). For the planned multiple linear regression analysis, assuming a medium effect size (f² = 0.30), a two-sided significance level (α) of 0.05, a power of 0.86, and nine independent predictors, the minimum required sample was 64 participants. To allow for potential attrition and incomplete data while maintaining adequate statistical power, the recruitment target was increased to 84 participants.

### Outcome measuress and procedures

Demographic and clinical information was obtained from participants and their parents or guardians using a data-collection form developed for this study. The demographic variables included the child’s gender, age, and educational status; parental age, education, and employment; marital status; primary caregiver; family size; and family income. Parental education was categorized as university, high school, middle school, preparatory school, or no formal education. Monthly family income was categorized as 0–9,000 Saudi riyals (SR), 10,000–14,000 SR, 15,000–24,000 SR, or ≥ 25,000 SR. Family size was categorized as fewer than six members or six or more members. Cancer-related variables included family history of cancer, leukemia type, time since diagnosis, and treatment regimen. Information reported by parents was cross-checked against the children’s medical records. The primary caregiver was categorized as either a family caregiver (a parent or another immediate family member) or another caregiver (e.g., a nanny or a nonfamily individual with primary responsibility for the child’s daily care). HRQoL was assessed using the Pediatric Quality of Life Inventory™ (PedsQL™) 3.0 Cancer Module. Upper- and lower-extremity function was assessed using the QuickDASH and the LEFS, respectively.

### Pediatric quality of life inventory™ 3.0 cancer module

The PedsQL™ 3.0 Cancer Module is a multidimensional instrument developed by Varni et al. [14] to assess the effects of disease and treatment on HRQoL in children with cancer. It is widely used in pediatric cancer research and is responsive to disease-specific interventions [21]. The Arabic version has demonstrated acceptable validity and reliability. The PedsQL™ 3.0 Cancer Module is available in age-specific versions for children and adolescents aged 5–7, 8–12, and 13–18 years. In accordance with the instrument guidelines, the parent-proxy version was used for children aged 5–7 years and was completed by a parent or primary caregiver [22]. The scale comprises 27 items across eight domains: pain and hurt (2 items), nausea (5 items), procedural anxiety (3 items), treatment anxiety (3 items), worry (3 items), cognitive problems (5 items), perceived physical appearance (3 items), and communication (3 items). Items are rated on a 5-point Likert scale, with higher transformed scores indicating fewer difficulties related to the disease or its treatment. The appropriate age-specific questionnaire was used for each participant [23]. Total scores > 80 were classified as high HRQoL, scores from 60 to < 80 as moderate HRQoL, and scores < 60 as low HRQoL [24].

### Physical function measures

#### Quick disabilities of the arm, shoulder and hand questionnaire

Children with leukemia may experience treatment-related muscle weakness, fatigue, and neuromuscular impairments that affect upper-extremity function and make everyday activities more difficult, potentially reducing HRQoL [25]. The QuickDASH is an 11-item questionnaire that assesses difficulty performing upper-extremity activities of daily living and the effects of upper-extremity disease or injury [26]. Items are rated on a 5-point Likert scale, with higher scores indicating greater disability [27]. The Arabic QuickDASH has demonstrated validity, reliability, and responsiveness in adults with upper-extremity musculoskeletal disorders [28–30]. Recent evidence also supports the construct validity and internal consistency of the QuickDASH in pediatric patients with upper-extremity conditions, supporting its use to assess upper-limb disability in school-aged children [31].

#### Lower extremity functional scale

The LEFS, developed by Binkley et al., is a 20-item self-reported, region-specific questionnaire used to assess lower-extremity functional status [32, 33]. It is quick and easy to complete and score and is feasible for use in most clinical settings. The LEFS has been validated in several languages and has demonstrated good content and construct validity, including in Arabic-speaking populations. Item scores are summed to yield a total score ranging from 0 to 80, with higher scores indicating better lower-extremity function [34–37].

### Statistical analysis

Analyses were conducted using IBM SPSS Statistics for Windows, version 19.0 (IBM Corp., Chicago, IL, USA). Continuous demographic and cancer-related variables were summarized using means and standard deviations, and categorical variables were summarized using frequencies and percentages. Before analysis, the data were checked for completeness. No participants were excluded because of missing data, and all PedsQL™ questionnaires were complete; therefore, no imputation was required. Domain and total scores were calculated according to the standard PedsQL™ scoring guidelines. HRQoL domains were summarized using the mean and standard deviation and the median and range. Domain scores were calculated as the sum of completed item scores divided by the number of completed items. Items were reverse-scored and linearly transformed to a 0–100 scale (0 = 100, 1 = 75, 2 = 50, 3 = 25, and 4 = 0), such that higher scores indicated better HRQoL. In accordance with the scoring guidelines, a domain score was not calculated when more than 50% of its items were missing. Pearson correlation coefficients were calculated for continuous variables, whereas Spearman correlation coefficients were calculated for categorical or ordinal variables to examine associations between total and domain-specific HRQoL scores and demographic, cancer-related, and physical-function variables. The normality of continuous variables was assessed using the Shapiro–Wilk test and visual inspection of histograms and Q–Q plots. Before multiple linear regression, the assumptions of linearity, independence of errors, homoscedasticity, normality of residuals, and absence of multicollinearity were evaluated. Linearity and homoscedasticity were assessed using scatterplots of standardized residuals against predicted values. Independence of errors was assessed using the Durbin–Watson statistic, and residual normality was evaluated using histograms and normal Q–Q plots. Multicollinearity was assessed using tolerance values and variance inflation factors (VIFs). All assumptions were considered adequately met before the final analyses were performed.

A theory-driven multiple linear regression analysis was performed using the enter method to identify factors independently associated with HRQoL. Predictor variables were selected on the basis of evidence from the pediatric oncology literature. The model included demographic characteristics (age group, gender, and child educational level), clinical characteristics (leukemia type, cancer therapy, and time since diagnosis), socioeconomic status (family income), and functional status measured using the QuickDASH and LEFS. All selected variables were retained in the model regardless of statistical significance to obtain adjusted estimates of their independent associations with HRQoL. Multicollinearity was assessed using the VIF, with values < 5 indicating no problematic multicollinearity [38, 39].

## Results

### Demographic and cancer- related characteristics of participants

Table 1 summarizes the demographic and cancer-related characteristics of the participants. The study included 86 children aged 5–14 years; 65.1% were aged 8 years or older. More than half of the participants (52.3%) were girls, and 79.1% were enrolled in nursery or school.

**Table 1:** Demographic and clinical characteristics of study participants (*n* = 86)

Variables	Category	*N* (%)
Gender	Boys	41 (47.7)
	Girls	45 (52.3)
Age	5–7 years	30 (34.9)
	8–14 years	56 (65.1)
Patient education	Yes (nursery/school)	68 (79.1)
	No	18 (20.9)
Marital status	Married	57 (66.3)
	Divorced/Widowed	29 (34.7)
Father age	≤ 45 years	59 (68.6)
	> 45 years	27 (31.4)
Mother age	≤ 45 years	77 (89.5)
	> 45 years	9 (10.5)
Education status of parents	Yes	70 (81.4)
	No	16 (18.6)
Father occupation	Employed	67 (77.9)
	Non-employed	19 (22.1)
Mother occupation	Employed	25 (29.1)
	Non-employed	61 (70.9)
Caregiver	Family (parents/siblings)	69 (80.25)
	Other caregivers (extended relatives or hired caregivers)	17 (19.75)
Family size	≤ 6	38 (44.2)
	> 6	48 (55.8)
Income	≤ 14,000 SR	60 (69.77)
	> 14,000 SR	26 (30.23)
Cancer related characteristics		
Cancer types	ALL	68 (79.0)
	AML	18 (21.0)
Time since diagnosis	≤ 12 months	41 (47.7)
	> 12 months	45 (52.3)
Current therapy	Yes	86 (100)
	No	0 (0)
Cancer treatment	CT	28 (32.6)
	CT + RT	47 (54.6)
	CT + RT + T	11 (12.8)
Family history of cancer	Yes	24 (27.91)
	No	62 (72.09)

*ALL* Acute Lymphoblastic Leukemia, *AML* Acute Myeloid Leukemia, *CT* Chemotherapy, *RT* Radiation Therapy, *T* Transplantation Therapy, *CT + RT* Chemotherapy and Radiation Therapy, *CT + RT+T* Chemotherapy and Radiation–Transplantation Therapy, *N* Number of participants, *SR* Saudi Riyal

Regarding parental characteristics, most fathers (62.8%) were 30–45 years of age, 81.4% had received formal education, and 77.9% were employed. Most participants (79.0%) had acute lymphoblastic leukemia (ALL), all were receiving treatment, and 27.9% had a family history of cancer. More than half of the children (52.3%) had been diagnosed more than 12 months previously. Regarding treatment, 54.6% were receiving chemotherapy and radiotherapy, 32.6% were receiving chemotherapy alone, and 12.8% were receiving chemotherapy, radiotherapy, and transplantation therapy.

Table 2 presents the HRQoL total and domain scores measured using the PedsQL™ 3.0 Cancer Module. Overall HRQoL was classified as high in 16.3% of participants, moderate in 58.1%, and low in 25.6%. The mean overall HRQoL score was 62.73 ± 16.47, indicating moderate HRQoL. Mean domain scores ranged from 43.52 to 71.12; procedural anxiety had the lowest score (43.52 ± 30.89), and communication had the highest (71.12 ± 30.79).

**Table 2:** Health-related quality of life and domain scores of the study participants (*n* = 86)

PedsQL-Cancer Modules	Mean ± SD	Median (IQR)
Pain and hurt	62.74 ± 27.19	63 (25)
Nausea	60.36 ± 24.60	50 (32)
Procedural Anxiety	43.52 ± 30.89	50 (50)
Treatment Anxiety	59.50 ± 29.96	59 (49)
Cancer Worry	62.64 ± 27.48	67 (35)
Cognitive Problems	68.93 ± 19.39	70 (36)
Perceived Physical Appearance	70.92 ± 23.45	75 (44)
Communication	71.12 ± 30.79	75 (50)
Total-HRQOL	62.7 ± 16.47	63 (26)

*PedsQL* Pediatric Quality of Life Inventory, *HRQOL* Health-Related Quality of Life, *SD* Standard deviation, *IQR* Interquartile Range (25th–75th percentile)

Table 3 presents the associations of sociodemographic and clinical characteristics with HRQoL. Girls had significantly higher total HRQoL and most domain scores than boys (all *p* < 0.05), except for the treatment-anxiety domain. Younger children, particularly those younger than 7 years, had significantly lower procedural-anxiety scores than older children (*p* < 0.001), indicating greater procedural anxiety. Children with higher educational attainment had higher total HRQoL and significantly better scores in the pain and hurt, nausea, treatment anxiety, cancer worry, and perceived physical appearance domains (*p* < 0.05). Higher parental educational attainment was associated with better total HRQoL and higher procedural-anxiety and cognitive-problems domain scores, whereas parental employment, maternal age, and family history of cancer were not significantly associated with HRQoL.

**Table 3:** Association between sociodemographic and clinical characteristics and quality of life of patients (*n* = 86)

Variables	*N*	Pain and hurt	Nausea	Procedural Anxiety	Treatment Anxiety	Cancer Worry	Cognitive Problems	Perceived Physical Appearance	Communication	Total HRQOL
	86	62.74 ± 27.19	60.36 ± 24.60	43.52 ± 30.89	59.50 ± 29.96	62.64 ± 27.48	68.93 ± 19.39	70.92 ± 23.45	71.12 ± 30.79	62.7 ± 16.47
Gender										
Boys	41	56.76±27.37	54.41±25.28	36.83±30.79	53.29±27.95	52.51±26.86	60.68±16.82	60.56±23.55	61.98±28.98	54.88±12.83
Girls	45	68.20±26.14^ᵵ^	65.78±22.91^ᵵ^	49.62±30.79^ᵵ^	65.16±30.89	71.87±24.89^±^	76.44±18.64^±^	80.36±19.14^±^	79.44±30.32^±^	69.89±16.26^±^
Patient age										
5-7 years	32	65.83±30.43	59.33±27.91	27.77±17.53	59.90±34.92	67.73±26.55	65.60±17.02	71.67±20.48	71.60±34.81	60.97±17.34
8-14 years	54	61.96±24.41	60.91±22.89	51.96±34.92^±^	59.29±27.28	59.91±27.82	70.71±20.48	70.52±27.35	70.86±28.76	63.68±16.07
Patient education										
Yes	68	70.13±23.69^±^	66.25±22.72^±^	50.62±29.86^±^	61.75±30.68	65.25±28.61	70.44±18.90^*^	71.50±18.89^*^	76.83±22.04	66.15±15.62^±^
No	18	36.11±19.59	38.11±18.17	16.72±7.29	51.00±26.08	52.78±20.49	63.22±20.68	59.22±18.62	69.60±32.69	49.83±3.14
Father age										
≤ 45 years	59	66.37±26.55	60.17±24.72	39.25±30.84	56.32±31.73	60.05±26.74	68.86±19.37	69.63±25.20	68.05±33.434	61.36±16.12
> 45 years	27	55.67±25.99	60.78±24.80	52.85±29.44 ^ᵵ^	66.44±24.79	68.30±28.72	69.07±19.79	73.74±19.19	77.81±23.23	65.74±17.12
Parents Education										
Yes	70	65.44±24.51	62.14±24.22	47.51±30.66^*^	59.97±30.59	64.44±25.84^±^	71.64±18.38^±^	72.13±22.80	72.37±30.68	64.70±15.65^*^
No	16	52.38±33.57	52.25±25.39	26.06±16.34	57.44±27.88	54.75±31.55	57.88±21.39	65.64±26.21	65.63±31.73.	54.13±17.69
Father occupation										
Employed	67	61.85±26.08	58.60±24.18	42.81±30.11	59.84±29.53	63.70±25.62	70.6618.70	71.75±23.14	73.75±30.09	63.10±16.14
Non-employed	19	65.89±31.38	66.58±25.71	46.05±34.28	58.32±32.21	58.89±33.74	62.84±21.01	68.00±24.91	61.84±32.27	61.42±17.97
Mother age										
≤ 45 years	77	63.06±26.89	58.71± 23.57	42.32± 30.77	58.34±29.38	61.95±26.73	68.96±19.99	70.99±22.74	70.97±30.53	62.10±16.05
> 45 years	9	62.56±26.46	74.44±30.046	53.78±31.87	69.44±34.74	68.56±34.45	68.67±13.95	70.33±30.41	72.33±34.95	68.11±19.92
Mother occupation										
Employed	25	55.60±24.44	54.64±22.47	47.64±30.61	61.08±31.91	59.36±29.99	74.92±16.37	73.60±23.14	72.04±28.28	62.76±15.41
Non-employed	61	66.05±27.17	62.70±25.22	41.84±31.11	58.85±29.37	63.98±26.52	66.48±20.10	69.82±23.67	70.74±31.98	62.72±17.00
Marital status										
Married	57	68.04±25.41^*^	65.26±23.34^**±**^	46.51±29.67	65.47±29.22^±^	66.96±26.11^ᵵ^	69.56±17.27	74.11±23.69	76.44±28.93^*^	66.54±14.22^*^
Divorced/Widowed	29	53.14±26.82	50.72±24.54	37.66±32.91	47.76±28.30	54.14±28.55	67.69±23.28	64.66±22.01	60.66±32.16	55.24±18.20
Caregiver										
Family	69	66.20±26.21^*^	63.26±25.01*	46.87±31.72^*^	60.23±31.19	66.20±27.09^*^	71.83±18.96*	72.48±23.86	72.93±32.35	65.28±16.32^*^
Other	17	50.06±25.39	48.59±19.32	29.94±23.49	56.53±24.95	48.18±24.83	57.18±16.99	64.59±21.16	63.76±22.83	52.41±12.96
Family size										
≤ 6	38	72.42±26.46^±^	68.82±27.8^±^	49.16±34.8	68.55±29.86^*^	73.24±24.81^±^	75.42±18.52^±^	76.76±24.23^*^	72.11±33.983	69.82±17.48^*^
> 6	48	55.56±24.68	53.67±19.54	39.06±26.9	52.33±28.33	54.25±26.80	63.79±18.67	66.29±21.95	70.33±28.372	57.13±13.31
Income										
≤ 14,000 SR	60	61.52±28.74	61.35±27.04	41.42±31.29	60.97±31.01	65.17±27.22	66.83±20.01	71.53±23.90	71.23±32.85	62.72±17.95
> 14,000 SR	26	66.46 ±21.37	58.08±18.04	48.38±29.98	56.12±27.67	56.81±27.72	73.77±17.27	69.50±22.76	70.85±26.06	62.77±12.74
Cancer-related characteristics										
Cancer types										
ALL	68	66.26 ± 25.42^±^	64.79 ± 23.46^±^	50.03 ± 29.73^±^	64.72± 28.51^±^	69.78 ± 24.59^±^	72.12 ± 18.25^±^	71.91 ± 23.64	71.81 ± 30.78	66.69± 15.37^±^
AML	18	50.72 ± 28.55	43.61 ± 21.46	18.94 ± 21.94	39.78 ± 27.63	35.67 ± 20.44	56.89 ± 19.29	67.17 ± 22.96	68.50 ± 31.63	47.78 ± 11.23
Treatment duration										
≤ 12 months	41	58.29±25.22	52.59±24.04	36.20±30.30	46.59±28.54	56.17±25.03	66.78±20.24	64.46±22.03	67.71±31.74	56.46±15.08
> 12 months	45	67.31±27.54	67.44±23.15^±^	50.20±30.23^*^	71.27±26.38^±^	68.53±28.54^*^	70.89±18.58	76.80±23.37^*^	74.22±29.92	68.44±15.72^±^
Current treatment										
CT	28	68.39±26.36^*^	62.68±26.22^*^	47.36±29.52^*^	67.50±28.46^*^	69.93±27.87^*^	77.46±14.44^*±^	80.32±23.22^*^	81.86±26.75^*^	69.61±17.27^±^
CT+RT	47	62.34±27.76	63.09±24.19	45.02±30.33	58.85±30.33	62.28±26.79	67.02±19.29	67.19±23.67	65.77±33.50	61.87±13.65
CT+RT+T	11	50.09±24.41	42.82±14.36	27.36±34.48	41.91±26.19	45.64±23.47	55.36±22.26	62.91±15.99	66.64±22.07	48.91±17.20
F-history of cancer										
Yes	24	63.13±29.79	62.08±27.30	38.21±32.62	51.00±36.05	63.54±27.49	70.25±18.05	64.63±25.65	64.58±37.02	60.46±16.57
No	62	62.97±25.66	59.69±23.68	45.58±30.22	62.79±26.85	62.29±27.68	68.42±19.99	73.35±22.27	73.65±27.95	63.61±16.48
Physical function										
DASH										
< 29.5	54	69.31±26.27^**±**^	68.80±22.11^±^	55.09±28.55^±^	667.72±26.97^±^	69.78±25.14^±^	72.76±18.35^*^	75.15±22.39^*^	72.35±30.63	69.17±13.83^±^
≥ 29.5	32	51.66±25.42	46.13±22.21	24.00±24.45	46.63±30.02	50.59±26.97	62.47±19.65	63.78±23.79	69.03±31.46	51.88±14.93
LEFS										
< 55.5	50	55.62±27.01	53.12±21.63	35.68±27.69	54.70±29.08^±^	54.06±25.31^±^	64.44±18.78^*^	69.82±22.31	75.50±26.53	57.98±14.42
> 55.5	36	72.64±24.51^**±**^	70.42±25.22^**±**^	54.42±32.18^**±**^	66.17±30.28^**±**^	74.56±26.19	75.17±18.72	72.443±25.17	65.03±35.39	69.33±17.04^±^

*N *Number of participants, *SD *Standard Deviation, *HRQOL *Health-Related Quality of Life, *PedsQL-Cancer Module *Pediatric Quality of Life Inventory, *ALL *Acute Lymphoblastic Leukemia, *AML *Acute Myeloid Leukemia, *CT *Chemotherapy, *RT *Radiation Therapy, *T *Transplantation Therapy, Physical Function

^ᵵ^ significant (*P *<0.05), ^*^ significant (*p*<0.01), ^±^Significant (p<0,001)

Children from married families, those living in smaller households, and those cared for directly by family members had significantly better HRQoL (all *p* < 0.01). Participants with acute lymphoblastic leukemia (ALL) had significantly higher total HRQoL and most domain scores than those with acute myeloid leukemia (AML) (all *p* < 0.001), except for perceived physical appearance and communication. Children diagnosed more than 12 months previously and those receiving chemotherapy alone reported significantly better HRQoL across most domains than their counterparts (all *p* < 0.001). Better physical function, as assessed using the QuickDASH and LEFS, was significantly associated with higher total HRQoL and most domain scores; however, the LEFS was not significantly associated with the communication domain.

### Predictors of quality of life among children with leukemia

Table 4 presents the results of the theory-driven multiple linear regression analysis performed using the enter method. The complete model explained 70.1% of the variance in total HRQoL (R² = 0.701; adjusted R² = 0.665; F(9, 76) = 19.753; *p* < 0.001). Significant independent predictors of total HRQoL were gender (β = 0.270, *p* < 0.001), age group (β = 0.229, *p* = 0.002), child educational level (β = 0.239, *p* = 0.001), cancer therapy (β = −0.211, *p* = 0.003), and lower-extremity function measured using the LEFS (β = 0.307, *p* < 0.001). Time since diagnosis (β = 0.096, *p* = 0.164), leukemia type (β = −0.133, *p* = 0.079), family income (β = −0.078, *p* = 0.233), and upper-extremity function measured using the QuickDASH (β = −0.067, *p* = 0.373) were not significant independent predictors of HRQoL.

**Table 4:** Multivariate model investigating independent predictors of PedsQL-Cancer domain scores (*n* = 86)

HRQOL Domain	Independent Predictors	β	R2	Adj. R2	F (sig)
	Gender	0.15			
	Age	0.25			
	Patient education	0.29			
Total HRQoL	Caregivers	-0.17	0.78	0.74	18.24 (*p* < 0.001)
	Family size	-0.18			
	Cancer treatment	-0.20			
	DASH	-0.20			
	LEFS	0.17			
Pain and Hurt	Patient education	0.21	0.33	0.26	4.24 (*p* < 0.001)
	Cancer duration	0.24			
Nausea	LEFS	0.20	0.41	0.30	6.59 (*p* < 0.001)
	Family size	-0.19			
Procedural Anxiety	Patient age	0.37	0.52	0.44	6.59 (*p* < 0.001)
	Caregivers	-0.20			
	LEFS	0.20			
Cancer Worry	Cancer type	-0.32	0.55	0.45	6.41 (*p* < 0.001
	LEFS	0.29			
Treatment Anxiety	Cancer duration	0.25	0.40	0.31	4.05 (*p* < 0.001)
	Gender	0.23			
	Patient education	0.33			
Cognitive Problems	Family income	0.20	0.50	0.42	6.05 (*p* < 0.001)
	Cancer type	-0.21			
	Cancer treatment	-0.21			
Perceived Physical Appearance	Gender	0.29	0.33	0.24	3.71 (*p* < 0.001)
	Gender	0.23			
Communication		0.30	0.33	0.25	4.16 (*p* < 0.001)
Parent education					
Caregivers		-0.23			
DASH		-0.32			

*HRQoL* Health-Related Quality of Life, *β* Standardized Beta Coefficient, *R2* Coefficient of Determination, *Adj. R2* Adjusted Coefficient of Determination, *F(sig)* F-statistic (Significance, *DASH* Disabilities of the Arm, Shoulder, and Hand (Quick-DASH), *LEFS* Lower Extremity Functional Scale

The regression model for the pain and hurt domain was statistically significant (F = 5.339, *p* < 0.001) and explained 38.7% of the variance (R² = 0.387). The LEFS score was the only significant independent predictor (β = 0.537), indicating that better lower-extremity function was associated with a higher pain and hurt domain score.

The regression model for the nausea domain was statistically significant (F = 4.643, *p* < 0.001) and explained 35.5% of the variance (R² = 0.355). The LEFS was the only significant predictor (β = 0.307), indicating that better lower-extremity function was associated with a higher nausea-domain HRQoL score.

The regression model for the procedural-anxiety domain was statistically significant (F = 8.886, *p* < 0.001) and explained 51.3% of the variance (R² = 0.513). Significant independent predictors were age (β = 0.211) and educational level (β = 0.427), with older age and a higher educational level associated with higher domain scores and therefore less procedural anxiety.

The regression model for the cancer-worry domain was statistically significant (F = 7.166, *p* < 0.001) and explained 45.9% of the variance (R² = 0.459). Gender (β = 0.219) and LEFS score (β = 0.278) were positively associated with the domain score, whereas leukemia type was a significant negative predictor (β = −0.342), indicating that cancer-related worry differed by leukemia type.

The regression model for the treatment-anxiety domain was statistically significant (F = 5.286, *p* < 0.001) and explained 38.5% of the variance (R² = 0.385). Child educational level (β = 0.224) and time since diagnosis (β = 0.241) were significant positive predictors, suggesting that a higher educational level and a longer time since diagnosis were associated with less treatment-related anxiety.

The regression model for the cognitive-problems domain was statistically significant (F = 6.63, *p* < 0.001) and explained 44.0% of the variance (R² = 0.440). Gender was a positive predictor (β = 0.333), whereas cancer treatment (β = −0.276) and family income (β = −0.189) were significant negative predictors of the cognitive-problems domain score.

The regression model for the perceived physical appearance domain was statistically significant (F = 4.01, *p* < 0.001) and explained 32.5% of the variance (R² = 0.325). Gender was the only significant predictor (β = 0.323), indicating that perceived physical appearance scores differed by gender.

Finally, the regression model for the communication domain was statistically significant (F = 2.657, *p* = 0.010) and explained 23.9% of the variance (R² = 0.239). Parental educational level was the only significant independent predictor (β = 0.229), indicating that a higher parental educational level was associated with better communication-related HRQoL.

## Discussion

This study evaluated HRQoL among children with leukemia, the most common childhood cancer and a major contributor to the burden of pediatric malignancy. Overall HRQoL was moderate (62.73 ± 16.47). Domain scores varied considerably: procedural anxiety and treatment anxiety had scores below 60 and were therefore the most affected domains in this cohort. In contrast, perceived physical appearance and communication had the highest scores, indicating relatively better HRQoL and fewer difficulties in these domains.

Alnaim et al. reported a similar finding in a sample of 100 children with acute lymphoblastic leukemia assessed using the PedsQL™ 3.0 Cancer Module; the mean HRQoL score was 61.9 ± 18.9 [13]. However, other Saudi Arabian studies by Alabbas et al. [39] and Hegazy et al. [24] reported relatively good overall HRQoL among children with cancer, with total scores of 73.48 and 72.3, respectively. Higher total HRQoL scores have also been reported in studies from the United States [20, 40, 41], where subgroup scores ranged from 70.08 to 75.1, and among children with cancer in China (71.02) [42], Lebanon (72.75) [11], Argentina (73.2), and Sweden (80.6) [43].

Conversely, the total HRQoL score in the present study was higher than those reported in Pakistan [44] and the Gaza Strip [12] (42.07 and 48.55, respectively). Studies from Japan [45], Canada [16], and Egypt [46] reported overall scores of 60.3, 64.26, and 62.29, respectively, which were comparable to our findings. In addition, a large cross-sectional study of 2,628 patients with leukemia across 76 countries found that HRQoL differed significantly by leukemia subtype and sex, with the poorest outcomes among patients with acute subtypes [47]. These findings underscore the importance of considering population and disease characteristics when comparing HRQoL across studies.

The pattern of domain scores observed in this study was broadly similar to that reported in previous studies [11, 13]. Communication and perceived physical appearance had the highest scores, whereas procedural and treatment anxiety had the lowest. Recent literature has attributed this pattern to the cumulative psychological burden of repeated invasive procedures, including lumbar punctures, bone marrow aspirations, and intravenous access [48, 49]. These findings emphasize the importance of routinely assessing children’s social, behavioral, and psychological well-being as part of cancer care. They also highlight the need to prevent and manage anxiety associated with medical procedures. Procedural anxiety can be distressing for both children with cancer and their parents and may be driven by pain, fear, and an unfamiliar hospital environment [39].

Our findings are consistent with three previous Saudi Arabian studies in which communication scores were relatively high and procedural-anxiety scores were relatively low [13, 39]. Similar results were reported in an Egyptian study, in which worry, perceived physical appearance, and procedural anxiety were among the lowest-scoring domains [46].

Collectively, these findings suggest that the psychosocial burden of treatment and procedural interventions is associated with quality of life in pediatric oncology. Recent literature supports this association, indicating that child-centered psychological preparation and procedural-support programs may reduce treatment-related anxiety and improve HRQoL [50]. However, causal effects must be evaluated in interventional studies because they cannot be established using the cross-sectional design of the present study.

### Factors associated with HRQoL among children with leukemia

In the present study, several demographic and treatment-related characteristics were associated with HRQoL. Functional status, measured using the QuickDASH and LEFS, was also associated with overall HRQoL.

Older children and girls reported better HRQoL in this study. The gender-related finding differs from much of the existing literature. Several studies [10, 46, 51] have reported that female sex and younger age are associated with poorer HRQoL, and a 2023 registry study of long-term childhood cancer survivors similarly found poorer functional quality of life and a greater symptom burden among female participants [52]. The divergent pattern in our sample may partly reflect differences in study populations: our cohort comprised children receiving active treatment rather than long-term survivors, and the association between gender and HRQoL may vary by disease phase and time since diagnosis [53, 54]. Culturally influenced expectations regarding emotional expression in Saudi Arabia may also affect how boys and girls report illness-related distress [39]. Although parent-proxy reports were obtained for children aged 5–7 years in accordance with PedsQL™ guidelines, parent–child agreement was not evaluated. Differences in reporting between children and caregivers therefore cannot be excluded. The observed gender differences should be interpreted cautiously and considered hypothesis-generating until confirmed in larger studies that include both child self-reports and parent-proxy reports across all age groups. Larger family size was associated with poorer HRQoL, consistent with previous reports. In larger families, each child may receive less individual attention, cancer-related financial pressures may be greater, and caregiving demands may be distributed across more children; together, these circumstances may reduce the support available to the child receiving treatment [54, 55]. Mounir and Abolfotouh [56] and Choo et al. [8] reported that poorer HRQoL was associated with younger age and lower educational attainment. Older children generally have a greater capacity to understand their illness, communicate their concerns, and manage treatment demands, whereas younger children depend more heavily on caregiver support and may find repeated medical procedures particularly distressing and unpredictable [48, 53]. The association with caregiver status may partly reflect the established relationship between caregiver well-being and child outcomes: when caregivers are fatigued or under-resourced, their capacity to provide consistent emotional support may decline, with downstream effects on the child’s coping and reported HRQoL [57]. Previous studies have also suggested that HRQoL among children with leukemia may be influenced by ongoing cancer therapy [58], time since diagnosis [59], age at diagnosis [51, 58, 59], gender [16, 51, 60], educational level, financial status, and parental characteristics [39].

Among Saudi Arabian studies, Alabbas et al. [39], Hegazy et al. [24], and Alnaim et al. [13] reported no significant associations of most demographic or medical characteristics with overall or domain-specific HRQoL, although patient age was an exception in some analyses.

The QuickDASH and LEFS were used to assess upper- and lower-extremity function, respectively. Functional status was significantly associated with overall HRQoL (*p* < 0.01) and with the pain and hurt, procedural-anxiety, and communication domains (*p* < 0.01). Mean QuickDASH and LEFS scores suggested that participants generally retained the ability to perform daily activities with relatively minor functional limitations. These findings support the importance of physical function to overall HRQoL, because children with better physical function may experience fewer activity limitations and better emotional well-being. However, the cross-sectional design precludes conclusions about the direction of this association.

Chemotherapy and prolonged hospitalization can reduce muscle mass, cardiorespiratory fitness, and peripheral nerve function, contributing to fatigue, one of the most disabling consequences of treatment [61, 62]. As physical capacity declines, children may be less able to attend school, play with peers, or participate in daily activities that support emotional well-being, thereby compounding the psychological burden of the illness. A 2024 meta-analysis of 12 randomized trials found that structured exercise programs during ALL treatment improved endurance, functional mobility, and cancer-related fatigue [61], and a 2026 meta-analysis reported similar HRQoL benefits following physiotherapy among pediatric cancer survivors [63]. Whether comparable benefits extend to children receiving active treatment remains uncertain and warrants prospective investigation.

### Clinical implications

Recent studies indicate that structured physical rehabilitation and physiotherapy interventions can improve HRQoL among pediatric cancer survivors by enhancing strength, endurance, and social reintegration [61–64]. The present findings indicate that demographic characteristics and functional status are associated with overall HRQoL. Routine assessment of physical function using validated instruments such as the QuickDASH and LEFS could be incorporated into comprehensive care for children with leukemia to identify those who may benefit from rehabilitation referral. The effectiveness of routine assessment and referral pathways in improving HRQoL should be evaluated in prospective interventional studies.

The association between physical function and HRQoL may inform the development of targeted interventions for evaluation in future trials. These findings suggest that tailored multidisciplinary approaches to support HRQoL in this population warrant further investigation. The study contributes to the limited evidence on HRQoL among children with leukemia in the Saudi healthcare context and aligns with the well-being and prevention priorities of the Saudi Vision 2030 Health Sector Transformation Program.

### Limitations

Several limitations should be considered when interpreting these findings. First, the cross-sectional design precludes causal inference; therefore, it remains unclear whether reduced physical function contributes to poorer HRQoL or whether poorer HRQoL contributes to declining physical function. Second, participants were recruited by convenience sampling from a single tertiary referral center, King Faisal Specialist Hospital and Research Center. This may limit the generalizability of the findings to children treated in other settings and may have excluded families facing greater barriers to specialized care. Although the sample size exceeded the minimum identified by the a priori power analysis, it remained relatively modest for subgroup analyses and multivariable regression; smaller associations may therefore have gone undetected. The regression findings should be interpreted cautiously and confirmed in larger independent cohorts before being generalized to the broader population of children with leukemia. Treatment phase (e.g., induction, consolidation, or maintenance) was not recorded separately and could not be evaluated as a potential determinant of HRQoL. Future studies should examine the effects of specific treatment phases on patient-reported outcomes. HRQoL data combined child self-reports from participants aged 8–14 years with parent-proxy reports for those aged 5–7 years. Proxy and self-reports may diverge, particularly for internal, nonobservable domains such as anxiety and worry, which were also the lowest-scoring domains in this sample. Pooling the two reporting sources may therefore have influenced the estimates. Questionnaire-based assessments are also subject to recall and social-desirability bias. In addition, although the QuickDASH has demonstrated good validity in pediatric patients with upper-extremity conditions, it was not developed or specifically validated for children with leukemia; findings related to upper-extremity function should therefore be interpreted cautiously. The absence of a healthy comparison group also limits assessment of the extent to which the observed HRQoL impairments were specifically attributable to leukemia and its treatment. Finally, several factors known to influence HRQoL, including parental psychological well-being, detailed socioeconomic circumstances, treatment intensity, chemotherapy regimen, and treatment-related complications, were not included in the analysis. Residual confounding therefore cannot be excluded. Larger multicenter longitudinal studies with more diverse samples are needed to validate these findings and provide a more comprehensive understanding of factors associated with HRQoL among children with leukemia.

## Conclusions

In this cross-sectional study of 86 children in Saudi Arabia receiving active treatment for leukemia, overall HRQoL was moderate, and procedural anxiety and treatment anxiety were the most affected domains. Demographic characteristics, cancer treatment, and physical function were associated with HRQoL. Although the cross-sectional design precludes causal conclusions and the observed gender differences require confirmation, these findings provide insight into factors associated with HRQoL in this population. They also highlight the importance of addressing treatment-related anxiety and maintaining physical function as components of comprehensive supportive care for children with leukemia. By providing evidence from the Saudi healthcare setting, this study contributes to the limited regional literature and supports the goals of Saudi Vision 2030, particularly the Health Sector Transformation Program’s emphasis on patient-centered care and improved quality of life for children with chronic illnesses.

## Data Availability

The datasets used and/or analysed during the current study are available from the corresponding author upon reasonable request.
